# Gravity-Driven Ultrahigh-Speed Electrospinning for the Production of Ethyl Cellulose Fibers with Tunable Porosity for Oil Absorption

**DOI:** 10.1021/acssuschemeng.4c08259

**Published:** 2024-12-19

**Authors:** Qiangjun Hao, John Schossig, Tyler Davide, Adedayo Towolawi, Cheng Zhang, Ping Lu

**Affiliations:** † Department of Chemistry and Biochemistry, Rowan University, Glassboro, New Jersey 08028, United States; ‡ Chemistry Department, Long Island University (Post), Brookville, New York 11548, United States

**Keywords:** gravity-driven ultrahigh-speed electrospinning (GUHS-ES), ethyl cellulose, porous fibers, oil absorption, green technology

## Abstract

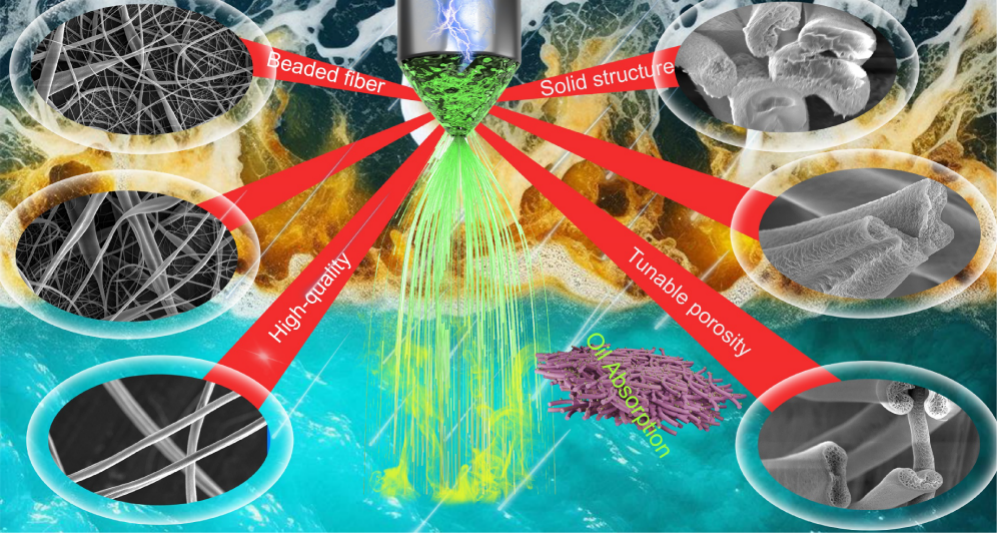

Ethyl cellulose (EC) is a biocompatible, renewable, and recyclable material with diverse sources, making it an attractive candidate for industrial applications. Electrospinning has gained significant attention for the production of EC fibers. However, conventional electrospinning methods face challenges such as bead formation, low yield, and the absence of porous internal structures, limiting both the functional performance and scalability. This study presents an optimized approach for producing EC fibers by using a gravity-driven ultrahigh-speed electrospinning (GUHS-ES) system. This system leverages gravity to reshape the Taylor cone morphology during electrospinning, enhancing stability and dramatically increasing throughput. As flow rates increase, the Taylor cone contracts inward, while the tip structure expands and stabilizes, reaching maximum size at ultrahigh flow rates (100–150 mL/h). This unique Taylor cone structure enables a fiber production rate of 24.5 g/h, hundreds of times greater than conventional electrospinning techniques. Another advantage of the GUHS-ES system is its ability to achieve both high diameter uniformity and adjustable porosity. At ultrahigh flow rates, the pore sizes of the EC fibers reached 321 nm. The highly porous structure of EC fibers exhibited an absorption capacity of 56.6 to 110.7 times their weight, exceeding most previously reported oil-absorbing materials and demonstrating high efficacy for rapid waste oil absorption. This green, efficient technology represents a promising advancement for the large-scale production and application of natural polymer fibers with broad implications for sustainable industrial processes.

## Abstract 2

This study introduces a sustainable, high-speed electrospinning technique for producing porous ethyl cellulose fibers for effective oil absorption.

## Introduction

Cellulose is the most abundant polysaccharide derived from renewable resources.^1−3^ It broadly exists in the cell walls of wood and plants, algal tissues, and the epidermal cell membranes of encysted animals.^4,5^ Ethyl cellulose (EC) is a linear polysaccharide derived from cellulose, consisting of cellulose backbones with partial replacement of hydrogen in cellulose hydroxyl end groups by ethyl end groups.^6^ Its high solubility in water and common organic solvents, low cost, biocompatibility, and biodegradability make it one of the most widely used cellulose derivatives.^7,8^ EC has been used in wearable sensing systems,^9,10^ energy storage,^11^ and biomedicine.^12^ EC polymer can be processed into various forms, including thin films,^13^ hydrogels,^14^ micro/nanoparticles,^15^ and micro/nanofibers.^16^ Among these, EC micro/nanofibers offer advantages over other EC forms due to their high surface area-to-volume ratio, excellent mechanical strength, and flexibility.^17^ They have been used in drug delivery,^18,19^ smart textiles,^20^ and conductive fabrics.^21^ Electrospinning is an eco-friendly and efficient method for the controlled synthesis of micro- and nanoscale fibers. Many works have addressed the electrospinnability of EC polymer.^22^ Nevertheless, challenges such as the formation of beaded fibers, low yield, and nonporous internal structure persist in electrospinning. Crucially, the average production rate of a lab-scale electrospinning process is around 0.1–0.2 g/h from a single spinneret, which dramatically limits the application of EC fibers.^8,23,24^

Extensive research efforts have been dedicated to overcoming the challenge of low productivity in electrospinning.^25,26^ To enhance electrospinning productivity, research has focused on advanced equipment designs, including multinozzle,^27^ needleless,^28^ and bubble electrospinning systems.^29^ Multinozzle electrospinning utilizes several nozzles to perform electrospinning simultaneously, and it is considered the most straightforward method to increase nanofiber productivity due to its ease of operation and high efficiency.^30^ However, this technique is technically costly and faces challenges such as significant jet interference, which hinders large-scale production of EC fibers.^31,32^ Additionally, cleaning multinozzle systems poses a significant challenge, as it requires large volumes of washing solvents, leading to increased environmental impact and higher operational costs. Needle-free electrospinning involves actively agitating the spinning solution while applying a high voltage. The interaction between the electric field and the agitated solution forms a Taylor cone on the surface, circumventing issues associated with multinozzle electrospinning.^31^

Needleless electrospinning enhances fiber productivity and reduces jet interference, but it faces significant challenges in practices, such as operational complexities, hard-to-produce advanced structures of nanofiber, and relatively higher voltage^33^ For instance, bubble electrospinning, an intriguing needleless technique for large-scale nanofiber production, suffers from excessive solvent evaporation driven by the air used to create bubbles.^34^ This not only contributes to environmental pollution but also results in costly solvent waste.^35,36^ Corona electrospinning is another needleless technique that eliminates an open liquid surface by continuously feeding the solution through a specialized nozzle, thereby reducing issues related to solution exposure.^30^ However, despite its advantages, this method faces several challenges: high throughput requires precise spinneret speed control to prevent overflow; the process demands extremely high voltages (up to 100 kV), increasing operational costs and safety risks; and the technology remains in its early research stages, with many parameters still needing optimization. Integrating electrospinning with other technologies is a common strategy to enhance productivity. Centrifugal or rotary jet electrospinning (CES) is an advanced method that combines electrostatic and centrifugal forces, facilitating scalability by increasing extrusion rates and enabling nanofiber collection in a 360° configuration.^37^ However, this approach requires optimizing two distinct sets of processing parameters, making it labor-intensive to set up and clean and posing challenges in achieving stable nanofiber production. Overall, current electrospinning techniques for nanofiber production have inherent limitations, highlighting the urgent need to develop greener, more sustainable electrospinning systems for the large-scale production of nanofibers.^38^

Our group recently developed a novel high-speed electrospinning technique utilizing sheath fluid and Taylor cone optimization, achieving remarkable control over the morphology, structure, and yield of EC fibers.^39^ This approach addresses the limitations of conventional electrospinning and broadens its potential applications. Although it significantly increased EC fiber production, yielding nearly 30 times the output of previous methods (∼4.48 g/h), the use of sheath fluid introduced concerns regarding environmental pollution and solvent waste. In this study, we optimized the uniaxial electrospinning technique for producing EC fibers without the use of a sheath fluid. This optimized technique is easy to operate and enhances both the yield and porosity of the EC fibers by leveraging gravity to reshape the Taylor cone. As the flow rate increases, the Taylor cone contracts inward toward the needle, while the tip structure expands outward. At a flow rate of 150 mL/h, the Taylor cone partially retracts into the needle, reaching the maximum tip expansion. This unique Taylor cone configuration enables EC fiber production at a rate of 24.5 g/h—hundreds of times greater than conventional electrospinning techniques. Furthermore, increasing the flow rate of the EC solution intensifies the stretching forces on the polymer, accelerating solvent evaporation and preventing the formation of beaded fibers, allowing precise control over the porous structure. The horizontal orientation of the electrospinning needle highlights the critical roles of gravity and electric field alignment in maintaining the Taylor cone stability. When gravity and electric forces are perpendicular, the stability of the Taylor cone and the resulting fiber morphology are largely dictated by the relative strengths of these opposing forces.

## Experimental Section

### Chemicals and Materials

Ethyl cellulose (EC) powder (9–11 mPa·s, 5% in 80:20 toluene/ethanol at 25 °C) was obtained from TCI America. Various oils, including motor oil, paraffin oil, silicone oil, olive oil, canola oil, and sunflower oil, were purchased from Amazon. Oil Red O and Sudan R dyes were sourced from BeanTown Chemical. All chemicals were used without further purification. The water used in the experiments was purified with a Millipore Direct-Q 8 UV water purification system, yielding a resistivity of 18.2 MΩ·cm at 25 °C. The uniaxial electrospinning needle (22 G) has an inner diameter of 0.40 mm, an outer diameter of 0.70 mm, and a needle tube length of 25 mm.

### Green Synthesis of EC Fibers with Tunable Porosity

EC fibers with controlled porosity were synthesized by using a green electrospinning method. A solvent mixture of ethanol and water (8:2 w/w) was employed to dissolve 20% w/w EC (molecular weight: 89 000 g/mol; viscosity: 9–11 mPa·s). The resulting EC solution was loaded into a metallic uniaxial spinneret equipped with a stainless-steel needle and electrospun at various flow rates (1, 10, 20, 30, 40, 50, 60, 100, and 150 mL/h) using programmable syringe pumps (Legato 110, KD Scientific), controlled through Adagio Syringe Pump Control Software (KD Scientific). A high-voltage DC power supply (ES30P-5W, Gamma High Voltage Research) applied 15 kV to the spinneret, initiating the formation of a Taylor cone and the subsequent ejection of a liquid jet containing the EC solution. The solvent evaporated rapidly as the fibers traveled toward a conductive collector positioned 20 cm beneath the needle. The entire process was conducted under controlled environmental conditions (25 ± 2 °C and 40 ± 3% relative humidity), maintained by the laboratory’s air conditioning system and an industrial-grade humidifier/dehumidifier in the fume hood. The collected EC fibers were dried in a vacuum oven at room temperature for 24 h before undergoing further analysis and characterization.

### Horizontal Electrospinning Setup to Investigate the Effect of Gravity on EC Fiber Morphology

To evaluate the influence of gravity on the formation of EC fibers, the uniaxial electrospinning device was reconfigured for horizontal operation. A 20% w/v EC solution (molecular weight: 89 000 g/mol; viscosity: 9–11 mPa·s) was fed into a stainless-steel spinneret at varying flow rates (1, 10, 20, 30, 40, 50, 60, 100, and 150 mL/h) to produce fibers. The electrospinning conditions, including the application of 15 kV to the spinneret, a collector positioned 20 cm from the needle, ambient temperature (25 ± 2 °C), and relative humidity (40 ± 3%), were consistent with the previously described setup. This horizontal configuration allowed for a direct comparison to the vertical setup, isolating the effect of gravity on the fiber deposition and morphology.

### Oil-Absorbing Properties of EC Fibers

The EC fibers were tested for their oil-absorbing capabilities by immersing them in an oil–water mixture for 30 s until saturation. After removal from the mixture, the mass of the oil-saturated EC fibers was recorded by using an analytical balance. To recover the absorbed oil, a basic evacuation device was employed, extracting the majority of the oil from the fibers for recycling purposes. Any residual oil loosely adhering to the outer surface of the fibers was removed by gently pressing the fibers between filter paper. The dried mass of the EC fibers was measured again, and the weight gain rate (*Q*_e_) was calculated using the following equation:
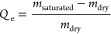
1where *m*_dry_ is the initial mass of the EC fibers and *m*_saturated_ is their mass after oil absorption. Each experiment was repeated three times to ensure accuracy, with error estimates derived from these trials.

### Characterization

The surface morphology and internal structure of the EC fibers were examined by using high-resolution field-emission scanning electron microscopy (SEM, Apreo S, FEI). To expose the fiber cross sections, the synthesized EC fibers were first frozen in liquid nitrogen at −195.8 °C for 10–20 min, followed by cutting with a sharp blade. Prior to SEM imaging, the samples were sputter-coated with a thin layer of gold for 60 s to enhance electrical conductivity. SEM images were captured at a working distance of 6 mm, with an accelerating voltage of 10 kV and a beam current of 0.40 nA. Fiber diameters were measured using ImageJ software (NIH), based on the representative SEM images. The crystalline structures of the EC fibers were analyzed by using X-ray diffraction (XRD) with a Bruker D8 Discover system, utilizing Cu Kα radiation at 40 kV and 40 mA. XRD data were collected in 0.02° steps, with a dwell time of 0.5 s per step, over a 2θ range of 5° to 90°. Fourier transform infrared (FTIR) spectroscopy was performed using the attenuated total reflection (ATR) method on a PerkinElmer Frontier spectrometer to assess the impact of the flow rate and solvent evaporation on the chemical composition of the EC fibers. Absorbance spectra were recorded in the wavenumber range of 4000–650 cm^–1^ with a spectral resolution of 4 cm^–1^, averaging 128 scans per sample. The specific surface area of the EC fibers was determined using the Brunauer–Emmett–Teller (BET) method in the relative pressure (*P*/*P*_0_) range of 0.05–0.35. Nitrogen desorption isotherm data were analyzed by using the Barrett–Joyner–Halenda (BJH) method to obtain the specific surface area values. Each measurement was performed in triplicate, and the average specific surface area was reported. Surface wettability and oil absorption properties were assessed by measuring the water and oil contact angles using a ramé-hart A-100 NRL contact angle goniometer equipped with a U5 series camera. The mean diameters and standard deviations of the EC fibers were calculated by analyzing over 100 individual fibers from the SEM images, with statistical analysis performed using OriginPro software (OriginLab).

## Results and Discussion

### Gravity-Driven Ultrahigh-Speed Electrospinning for the Green Production of EC Fibers with Tunable Porosity

**Figure 1:**
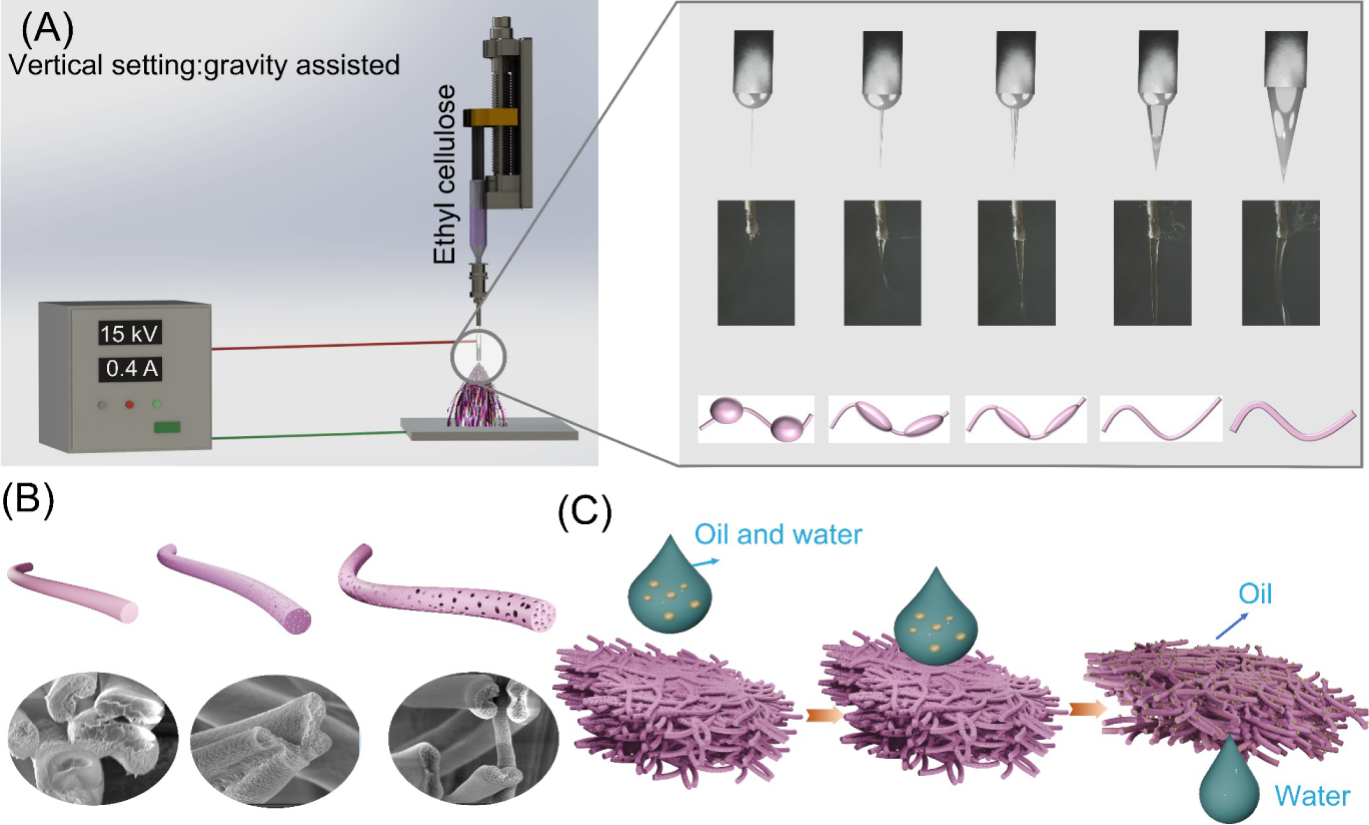
Schematic representation of GUHS-ES of EC fibers with tunable porosity for oil absorption applications. (A) Diagram of the GUHS-ES setup, illustrating the electrospinning process with gravity assistance. The right inset highlights the influence of gravity on the morphology of the Taylor cone and the formation of EC fibers at varying flow rates. (B) Synthesis of EC fibers with adjustable pore size by controlling the flow rate during electrospinning, as demonstrated in the SEM images. (C) Application of porous EC fibers for effective oil–water separation, where the fibers selectively absorb oil from an oil–water mixture.

Figure 1A illustrates the GUHS-ES process, which enhances both the production yield and the structural properties of EC fibers using a uniaxial electrospinning device (Figure 1A, left inset). By increasing the flow rate of the EC solution, the shape of the Taylor cone is transformed. Traditional electrospinning typically operates at a flow rate of ∼0.1–1 mL/h, producing a hemispherical Taylor cone with a fine tip.^40^ This slow flow rate not only limits the production speed but also increases the risk of clogging. At moderate increases (up to 10 mL/h), the Taylor cone became unstable, further aggravating clogging issues. However, a flow rate range of 20–60 mL/h stabilized the Taylor cone, causing it to contract toward the needle interior, while the tip expanded outward. At even higher flow rates (100–150 mL/h), the Taylor cone partially retracted into the needle, with the tip reaching maximum expansion. This distinctive Taylor cone geometry enables the production of EC fibers at a remarkable rate of 24.5 g/h from a single spinneret, a throughput that surpasses that of conventional electrospinning methods by several orders of magnitude. The stable high-speed jet exerts significant tensile forces on the EC polymers, preventing the formation of beads and promoting uniform fiber morphology (Figure 1A, right inset). Additionally, the ultrahigh flow rate accelerates solvent evaporation, leading to the development of porous fibers (Figure 1B). Crucially, the pore size of the EC fibers can be finely tuned by adjusting the flow rate, offering a high degree of control over the fiber porosity. The resultant porous, hydrophobic EC fibers, characterized by a high specific surface area, are highly suited for sustainable absorption applications. In this study, the porous EC fibers exhibited exceptional oil absorption capacities, along with excellent recyclability, making them ideal candidates for oil–water separation and other environmental applications (Figure 1C).

**Figure 2:**
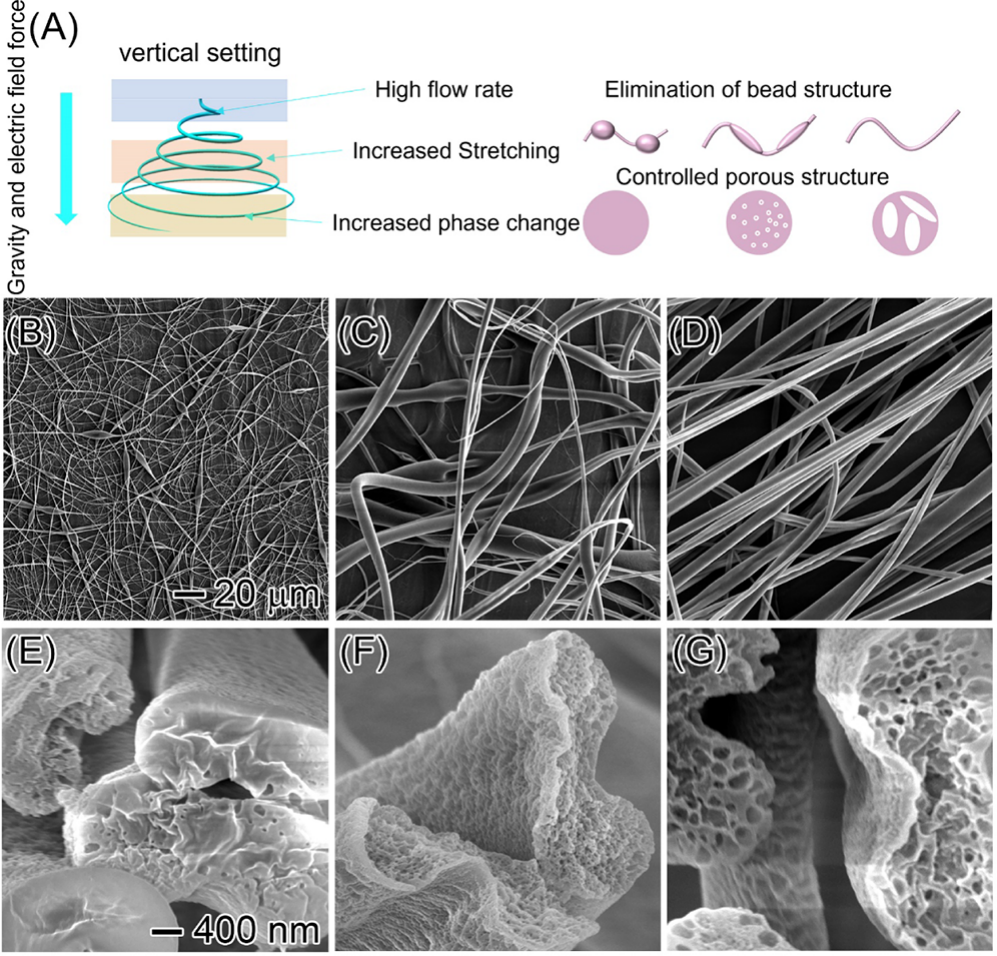
SEM images showing the morphology and porosity of EC fibers at different flow rates. (A) Schematic of the mechanism by which high EC flow rates promote fiber stretching and accelerate solvent (ethanol) phase separation: (B) 1, (C) 30, and (D) 150 mL/h. Cross-sectional SEM images corresponding to these flow rates: (E) 1 mL/h, showing a solid structure; (F) 30 mL/h, revealing the development of porous structures; and (G) 150 mL/h, highlighting a highly porous morphology. The 20 μm scale bar in (B) applies to images (B–D), and the 200 nm scale bar in (E) applies to images (E–G).

Electrospinning of EC fibers encounters three key challenges: clogging, low yield, and formation of beaded structures. Figure 2A illustrates the gravity-driven electrospinning mechanism used to achieve ultrahigh-speed production of the EC fibers. In our design, gravity and the electric field work in tandem, exerting downward forces on the EC polymer, generating substantial tensile stress that eliminates beaded structures, accelerates solvent evaporation, and yields fibers with tunable porosity. Figure 2B shows SEM images of EC fibers produced at a flow rate of 1 mL/h, where beaded structures and nonuniform diameters are prominent, consistent with previous studies.^41^ As the flow rate increases, the gravitational force exerted on the EC polymer intensifies, reducing the level of formation of beads, although the morphology remains inconsistent (Figure 2C). At an ultrahigh flow rate of 150 mL/h, the beaded structures are fully eliminated, and the fibers achieve uniform dimensions (Figure 2D). Additional flow rates of 10, 20, 40, 50, 60, and 100 mL/h were evaluated to systematically investigate the impact of the flow rate on EC fiber morphology (Figure S1). The results validate that increasing the gravitational influence enhances the uniformity of EC fibers, facilitating the production of fibers. It is important to note that a flow rate of 150 mL/h approaches the operational limit of our syringe pump due to the extreme pressure exerted. Further increases in the flow rate led to pump stalling and malfunction. However, theoretically, by employing high-pressure systems, such as high-pressure pumps and stainless-steel syringes, the production capacity of our GUHS-ES system could be significantly enhanced.

In addition to significantly improving the morphology of the EC fibers, adjusting the flow rate allows for measurable and reproducible control over their porosity, thereby enhancing their functional properties. As shown in Figure 2E, at a flow rate of 1 mL/h, the fibers exhibit a solid structure, consistent with previously reported findings.^42,43^ As the flow rate increases to 30 mL/h, porous structures begin to emerge (Figure 2F), and at an ultrahigh flow rate of 150 mL/h, the average pore size reaches a maximum of 321 nm (Figure 2G). A systematic analysis of the EC fiber structure at flow rates of 10, 20, 40, 50, 60, and 100 mL/h (Figure S1) demonstrates that the internal porosity of the fibers can be finely tuned by adjusting the flow rate. Additionally, a comparison of surface morphologies reveals that higher flow rates lead to rougher fiber surfaces (Figure S2) and higher internal porosity (Figure S3). This phenomenon can be attributed to two primary mechanisms related to the dynamics of the EC polymer solution. First, rapid solidification occurs at higher flow rates. If solvent evaporation is faster at the surface, while the core remains liquid, internal stresses are generated, contributing to surface roughness and pore formation. Second, the phase separation process is accelerated at higher flow rates. The faster ejection of the EC solution from the spinneret increases the rate of solvent–polymer separation, further contributing to surface roughness and pore formation.^44^ These findings support our hypothesis, significantly enhance the functionality of EC fibers, and expand their potential applications.

**Figure 3:**
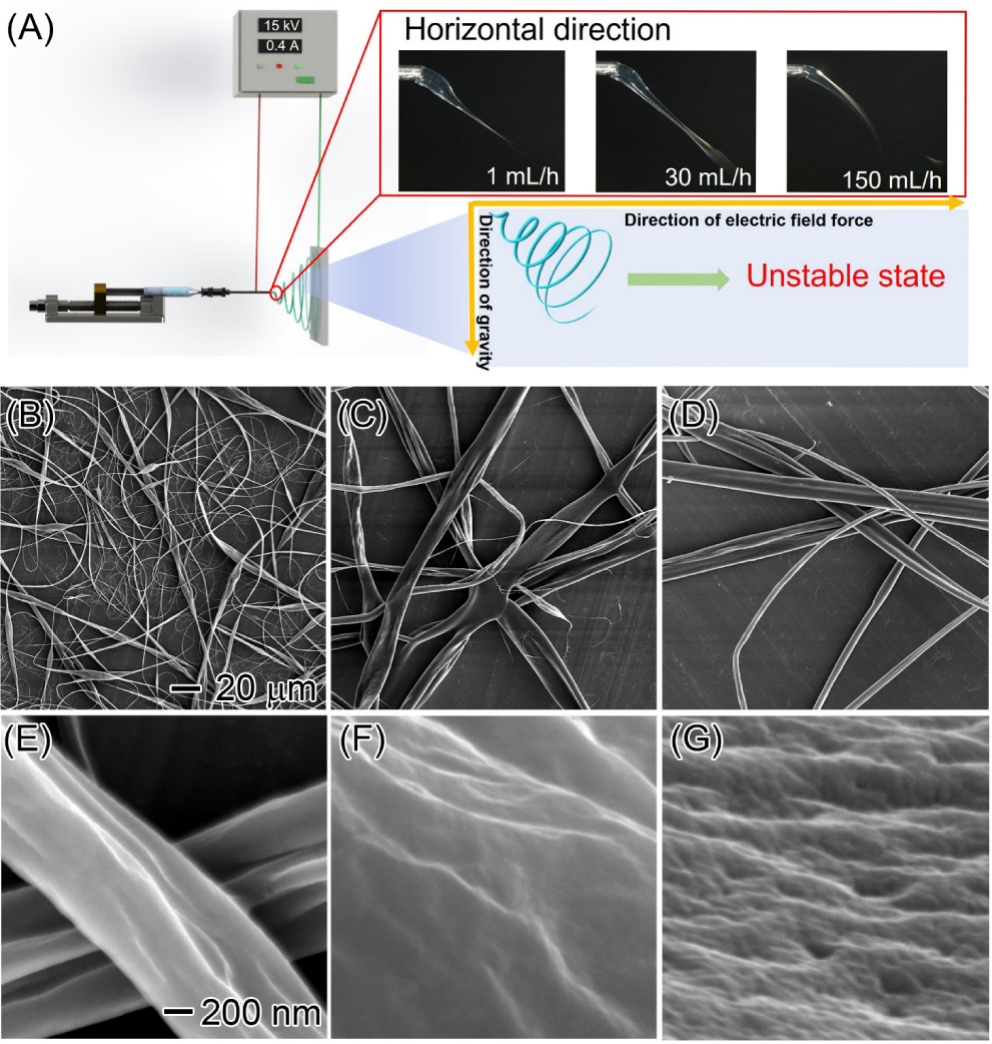
SEM images illustrating the effect of different flow rates on EC fiber morphology during electrospinning in a horizontal setup. (A) Schematic of the electrospinning process in the horizontal configuration (top right: microscope images of different EC flow rates; bottom right: mechanism of horizontal electrospinning). (B) SEM image showing the morphology of EC fibers at 1 mL/h, (C) at 30 mL/h, and (D) at 150 mL/h. (E–G) SEM images of the corresponding surface structures at flow rates of 1, 30, and 150 mL/h, respectively. The scale bar in (B) represents 20 μm and applies to (B–D), while the scale bar in (E) represents 200 nm and applies to (E–G).

These findings demonstrate that gravity plays a critical role in stabilizing the Taylor cone during electrospinning and optimizing the morphology of the EC fibers. To further explore the influence of gravity’s direction on the electrospinning process and the resulting fiber morphology, the equipment was reconfigured to a horizontal setup, allowing gravity to act downward while the electric field force was applied horizontally. Figure 3A illustrates the schematic of this configuration, where gravitational and electric field forces act perpendicular to one another. In this horizontal setup, gravity causes the EC polymer jet to deflect downward, while the electric field stretches it horizontally. The interplay between these two opposing forces strongly affects the stability of the Taylor cone (Figure 3A, right). At a low flow rate (1 mL/h), as shown in Figure 3B, the electric field dominates, allowing the Taylor cone to remain relatively stable. The resulting EC fibers, despite some beaded structures and uneven diameters, are similar to those produced in the vertical setup. As the flow rate increases, the influence of gravity grows, and when it becomes comparable to the electric field force, the Taylor cone becomes unstable, leading to significant agglomeration and morphological collapse of the fibers (Figure 3C). However, at an ultrahigh flow rate of 150 mL/h, EC fibers with uniform diameters were successfully produced (Figure 3D). Additional results at flow rates of 10, 20, 40, 50, 60, and 100 mL/h (Figure S4) further confirm that when gravity and the electric field are misaligned, the fiber morphology is determined by the relative strength of these forces. In conventional electrospinning setups, orientation (horizontal or vertical) has minimal impact on fiber morphology because, at low flow rates, the gravitational force is not strong enough to significantly influence Taylor cone stability.

The direction of gravity plays a critical role in shaping the surface morphology of the EC fibers. At a low flow rate of 1 mL/h, the fibers exhibit a smooth surface (Figure 3E). As the flow rate increases, unlike in the vertical setup, no porous structures form and the surface remains smooth (Figure 3F). However, at an ultrahigh flow rate of 150 mL/h, the surface becomes rough and porous structures begin to emerge (Figure 3G). Additional SEM images at flow rates of 10, 20, 40, 50, 60, and 100 mL/h further highlight the variability in surface morphology (Figure S5). These observations demonstrate that the alignment of the electric field and gravitational forces significantly influences the fiber surface structure. In the horizontal setup, greater force is required to overcome surface tension and facilitate solvent–polymer phase separation compared with the vertical configuration. In the horizontal orientation, the electric field force primarily balances the aerodynamic drag, whereas in the vertical (top-down) setup, both gravity and the electric field counteract the drag.^45^ These findings challenge the previous assumption that the orientation of the electrospinning setup does not significantly impact fiber synthesis. They also emphasize the critical role of gravity in supporting ultrahigh-speed electrospinning.

**Figure 4:**
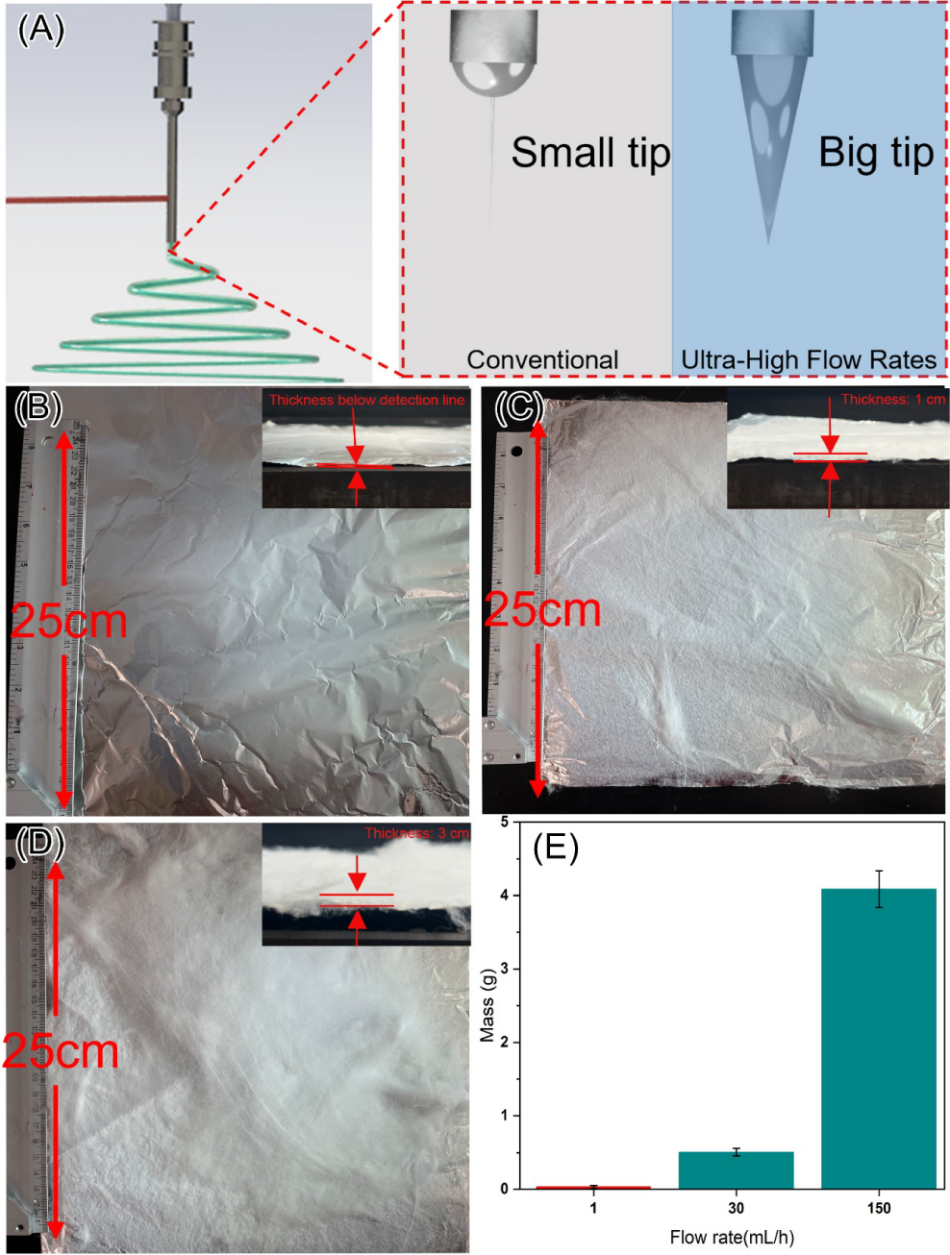
Comparison of EC fiber production at different flow rates over 10 min: (A) schematic illustrating the mechanism of ultrahigh-speed EC fiber production driven by gravity; (B) fiber production at 1 mL/h, with thickness below detection (inset); (C) fiber production at 30 mL/h, showing a thickness of 1 cm (inset); (D) fiber production at 150 mL/h, with a thickness of 3 cm (inset); (E) mass of EC fibers produced at flow rates of 1, 30, and 150 mL/h.

Extremely low yield is the primary challenge in producing fibers through electrospinning.^46^ Conventional electrospinning techniques for EC fibers typically operate at flow rates of ∼0.1–1 mL/h, resulting from a hemispherical Taylor cone with a tiny tip, which significantly limits the spinning speed. The slow spinning speed combines with rapid solvent evaporation when volatile solvents (e.g., ethanol) are used, increasing the concentration and surface tension of the EC solution and leading to clogging and instability in the electrospinning process. This study focuses on optimizing the Taylor cone shape through the effects of gravity, enabling ultrahigh-speed production of EC fibers. As illustrated in Figure 4A, reshaping the Taylor cone using gravity at higher flow rates significantly enhances fiber production. At ultrahigh flow rates, the Taylor cone contracts entirely within the needle, while the tip structure expands, leading to a dramatic improvement in the production yield of EC fibers. At a low flow rate of 1 mL/h, only a small amount of fibers (0.035 g) is produced after 10 min of electrospinning (Figure 4B). This low yield is primarily due to the rapid evaporation of the solvent, causing semisolid formation near the needle and disrupting the smooth progression of the electrospinning process.

At an increased flow rate of 30 mL/h, high-flow-rate electrospinning produces 0.51 g of EC fibers in the same 10 min period (Figure 4C), representing more than a 30-fold increase of the theoretical production rate compared to conventional electrospinning at 0.1–1 mL/h. Although this actual yield is slightly lower than the theoretical 30-fold increase, the discrepancy can be attributed to fiber loss during collection, as some fibers adhere to the chamber walls during rapid production. Ultrahigh-speed electrospinning at 150 mL/h further pushes the yield limit, producing 4.09 g of EC fibers in just 10 min (Figure 4D) from a single spinneret. This represents an over 100-fold increase compared to traditional methods (Figure 4E), demonstrating a significant advancement in single-needle electrospinning. The GUHS-ES technique thus provides an effective, scalable solution for producing large quantities of fibers from renewable polymers like EC. This method holds great promise for improving the efficiency of fiber production while maintaining uniformity.

The process yield is critical for evaluating the cost-effectiveness of a method, ensuring efficient material utilization and consistent product quality for industrial and research applications. In electrospinning, a high process yield highlights the feasibility of large-scale production. In this study, at low flow rates (1 mL/h), the throughput is extremely low, and calculating the process yield over a short duration (e.g., 10 min) is challenging due to the minimal mass of fibers produced. This results in a higher margin of error in the yield measurement. However, the fiber collection efficiency at this flow rate is typically high as the slower jet formation minimizes fiber loss to the chamber walls. At a moderate flow rate (30 mL/h), Taylor cone stability is poor, leading to intermittent jet formation and uneven fiber deposition. Additionally, the higher flow rate causes a significant amount of fibers to adhere to the chamber walls rather than be collected on the target substrate. As a result, the process yield was measured at approximately 51% of the theoretical solute mass. This highlights the impact of unstable cone formation and a suboptimal collection design at moderate flow rates. At ultrahigh flow rates (150 mL/h), Taylor cone stability improved significantly due to the optimized interplay between gravitational forces and the electric field. This resulted in continuous and consistent jet formation with a substantial increase in the amount of fibers collected on the substrate. The process yield at this flow rate was measured to be 81.8% of the theoretical solute mass, demonstrating the benefits of improved stability and high throughput. If we account for fibers that adhered to the side walls and other components of the setup, the total recovery of the theoretical solute mass would approach 100%. However, these fibers were not included in the primary process yield calculation, as they were not collected on the designated substrate. Redesigning the collector chamber to minimize fiber loss to nontarget surfaces could further enhance the process yield and enable nearly complete recovery of the solute.

### Effect of the Flow Rate on the Physical and Chemical Structure of EC Fibers

**Figure 5:**
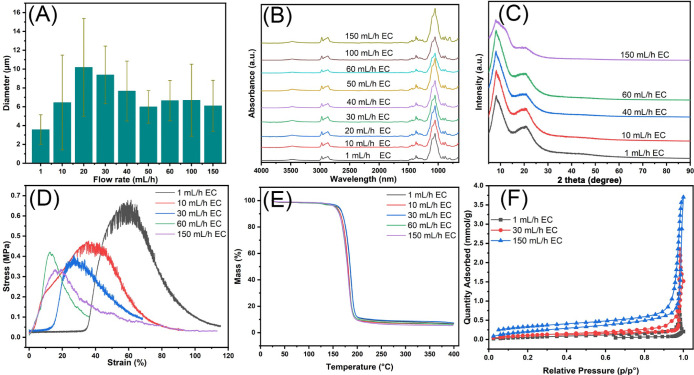
Comprehensive characterization of EC fibers produced at different flow rates. (A) The average diameter of EC fibers across various flow rates, highlighting the impact of increasing flow rates on fiber size. (B) FTIR spectra showing the chemical structure and functional group presence in EC fibers. (C) XRD profiles illustrating the crystalline structure of EC fibers at different flow rates. (D) Tensile strength versus strain curves for EC fibers, demonstrating the mechanical properties at various flow rates. (E) TGA data showing the thermal stability of EC fibers as a function of flow rates. (F) Nitrogen adsorption isotherms for EC fibers produced at 1, 30, and 150 mL/h flow rates, indicating differences in surface areas and porosity.

The diameters of EC fibers show a trend of initially increasing and then decreasing as the flow rate increases (Figure 5A). At relatively low flow rates (1–20 mL/h), the diameter of EC fibers increases from 3.56 ± 1.57 μm to 10.18 ± 5.19 μm as the flow rate rises, creating a distinct upward trend in fiber diameter as the flow rate increases from 1 mL/h to 20 mL/h. However, when the flow rate exceeds the threshold of ∼20 mL/h, the trend reverses. The fiber diameter decreases from 9.38 ± 3.05 μm to 6.09 ± 2.71 μm at ultrahigh flow rates (30–150 mL/h). The nonlinear relationship between the flow rate and fiber diameter in electrospinning is influenced by jet stability, solvent evaporation, and fiber stretching dynamics. Initial increase in diameter (1–20 mL/h): at low flow rates, increasing flow provides a larger volume of solution at the tip, which enlarges the Taylor cone and fiber diameter. The limited electrostatic force at these rates is not sufficient to stretch the fibers significantly, so the diameter increases with the flow rate.^47^ Decrease in diameter (20–50 mL/h): as the flow rate rises, jet velocity increases, intensifying stretching forces on the fiber. Rapid elongation and solvent evaporation dominate at this stage, resulting in thinner fibers as the diameter decreases. Stabilization of diameter (50–150 mL/h): at ultrahigh flow rates, EC fiber diameter stabilizes as the polymer supply increase no longer affects it. This balance between polymer ejection, stretching forces, stable solvent evaporation, and charge repulsion leads to consistent fiber diameter.^48^

FTIR spectroscopy is a valuable tool for analyzing intermolecular interactions in polymers by detecting peak shifts and assessing molecular compatibility. Figure 5B displays the FTIR spectra of EC fibers produced at various flow rates. At a low flow rate (1 mL/h), a broad band centered at 3475 cm^–1^ corresponds to the O–H stretching vibration. The peaks at 2974 and 2869 cm^–1^ are attributed to C–H stretching vibrations, while the peak at 1375 cm^–1^ is associated with C–H bending. Additionally, the band at 1052 cm^–1^ is due to C–O–C stretching. These assignments are consistent with previously reported spectra for EC.^11,21^ Despite the increase in the flow rate from 1 to 150 mL/h, the peak positions remain unchanged, indicating that the chemical structure of EC fibers is not affected by variations in the flow rate. To further investigate the impact of the flow rate on the crystalline properties of EC fibers, XRD analysis was conducted. Figure 5C presents the diffraction patterns of EC fibers at different flow rates, showing a sharp diffraction peak at 2θ = 7.9° and a broad peak at 2θ = 20.6°, which are characteristics of EC’s crystalline regions.^49,50^ These diffraction patterns indicate that the crystalline structure of the EC fibers remains stable across all flow rates. This suggests that while the flow rate influences the morphology of the fibers, it does not alter their underlying chemical structure or crystallinity.

The flow rate has a noticeable, though modest, impact on the mechanical properties of the EC fibers. At lower flow rates (1 and 10 mL/h), the fibers exhibit higher strength and stiffness (Figure 5D). As the flow rate increases (40 and 60 mL/h), the mechanical properties decline slightly. At an ultrahigh flow rate of 150 mL/h, the fibers become more flexible, with the lowest maximum stress of approximately 0.3 MPa at a strain of about 20%. This reduction in mechanical strength is primarily due to the porous structure of the fibers formed at high flow rates, which introduces a significant number of voids. These voids reduce the amount of solid material available to bear applied forces, thereby weakening the fibers’ ability to resist stress. Additionally, the presence of pores leads to discontinuities and irregularities in the fiber structure, creating weak points, where stress concentrates. These structural defects increase the likelihood of failure under a tensile load, leading to a decrease in tensile strength. Thus, the porosity in fibers contributes to structural weaknesses, reduces material density, and concentrates stress, ultimately diminishing their mechanical strength.

Figure 5E shows the TGA curves for EC fibers prepared at different flow rates (1, 10, 30, 60, and 150 mL/h). TGA analysis was conducted to examine the thermal stability of the fibers as a function of the flow rate. The results show that the mass of the EC fibers remains stable up to approximately 200 °C regardless of the flow rate. A sharp mass loss occurs between 200 and 300 °C, indicating the onset of thermal degradation. After 300 °C, the mass stabilizes, suggesting that most of the decomposition has been completed. Overall, the TGA analysis indicates that EC fibers produced at various flow rates exhibit similar thermal stability, with decomposition beginning at around 200 °C and ending after 300 °C.

The BET method was employed to measure the surface area and pore size of EC fibers produced at various flow rates. Figure 5F shows the nitrogen adsorption–desorption isotherms of EC fibers prepared at flow rates of 1, 30, and 150 mL/h. The isotherm for the fibers spun at 150 mL/h shows a sharp increase at a high relative pressure (*P*/*P*_0_ ≈ 1.0), characteristic of type IV isotherms, indicating the presence of mesopores. This suggests that EC fibers produced at ultrahigh flow rates possess a significant mesoporous structure. The adsorption capacity of fibers spun at 150 mL/h reached 3.5 mmol/g, corresponding to a BET surface area of 16.9 m^2^/g. This is significantly higher than the values observed for fibers produced at 30 mL/h (2.5 mmol/g, 7.9 m^2^/g) and 1 mL/h (1.5 mmol/g, 5.9 m^2^/g). These results demonstrate a substantial increase in both the adsorption capacity and specific surface area with an increasing flow rate, demonstrating the influence of the flow rate on enhancing the structural properties of EC fibers. The pore size distribution, calculated using Barrett–Joyner–Halenda (BJH) desorption analysis (Figure S6), shows the variation in pore volume (cm^3^/g) with pore width (nm) for fibers produced at different flow rates. The distribution shifts markedly with an increasing flow rate. Fibers produced at an ultrahigh flow rate of 150 mL/h exhibited a broader pore size distribution and larger pore volumes compared to those produced at lower flow rates. Specifically, the fibers spun at 150 mL/h displayed a peak pore size of around 80 nm, with a distribution ranging from 50 to 140 nm. In contrast, fibers spun at 30 mL/h showed a narrower distribution, peaking between 60 and 80 nm with an upper limit around 120 nm. Fibers produced at the lowest flow rate of 1 mL/h exhibited the smallest pore sizes with a relatively flat distribution and peak sizes below 50 nm. Pore volume also increased significantly with higher flow rates. The fibers spun at 150 mL/h exhibited the highest pore volume, peaking at approximately 0.124 cm^3^/g, while those spun at 30 mL/h reached 0.076 cm^3^/g. The fibers produced at 1 mL/h had the smallest pore volume, with a maximum of only 0.028 cm^3^/g (Table 1). This increase in the pore volume at higher flow rates can be attributed to the increased tension and enhanced phase separation during the electrospinning process. At higher flow rates, these effects result in larger, more uniform fibers with pronounced intrafiber voids, thereby increasing overall porosity. In contrast, fibers produced at lower flow rates are more prone to structural defects such as solid beads, leading to smaller pore sizes and reduced porosity.

**Table 1:** BET Analysis Results Detailing the Specific Surface Area, Total Pore Volume, and Mean Pore Diameter for EC Fibers Produced at 1, 30, and 150 mL/h

Sample	as,BET (m^2^g^–1^)	Total pore volume (*P*/*P*_0_ = 0.990) (cm^3^ g^–1^)	Mean pore diameter (nm)
1 mL/h	5.91	0.28	18.68
30 mL/h	7.87	0.076	26.09
150 mL/h	16.90	0.124	30.78

### Oil Absorption Performance of EC Fibers

**Figure 6:**
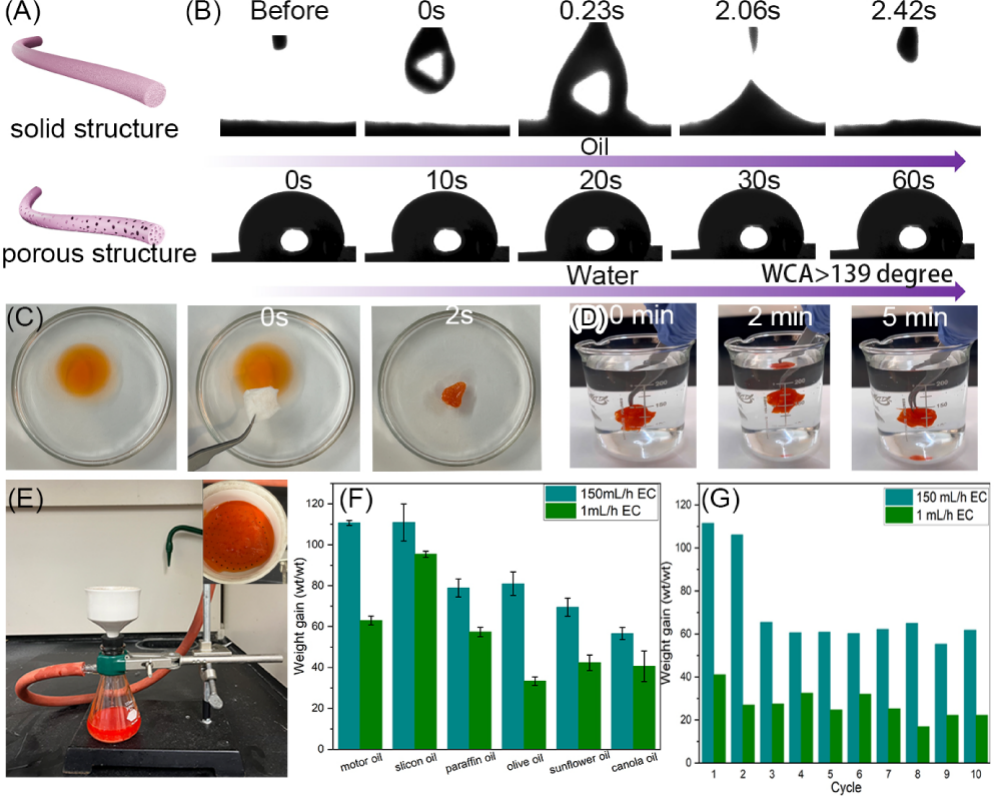
Oil absorption capabilities of hydrophobic EC fibers: (A) schematic diagrams illustrating the solid structure of EC fibers produced at 1 mL/h and the porous structure of those produced at 150 mL/h; (B) oil contact angle (OCA) measurements (top) and water contact angle (WCA) measurements (bottom), demonstrating the hydrophobicity of EC fibers with a WCA > 139°; (C) time-lapse images capturing the rapid absorption of motor oil by a small piece of porous EC fibers; (D) stability tests showing that absorbed motor oil is securely retained in the porous EC fibers, with no leakage even when submerged in water; (E) easy recovery of absorbed oil from EC fibers using a standard lab vacuum filtration setup; (F) oil absorption capacity of EC fibers for various types of oil; and (G) absorption cycling performance of EC fibers for motor oil, demonstrating their reusability over multiple cycles.

Both solid (low flow rate) and porous (ultrahigh flow rate) EC fibers (Figure 6A) were evaluated for their oil absorption properties. Figure 6B highlights the hydrophobic and oil-absorbing capabilities of the porous EC fibers. The results demonstrate that the porous fibers rapidly absorbed oil within 2 s, achieving an oil contact angle (OCA) of 0° (Figure 6B, top). Simultaneously, these fibers exhibited strong hydrophobicity, with a water contact angle (WCA) consistently around 139° (Figure 6B, bottom). The solid EC fibers showed similar OCA and WCA values, with an OCA of 0° and a WCA of 120.6° (Figure S7). This suggests that the porous structure does not significantly affect the oil and water contact angles, as these properties are largely determined by the physicochemical characteristics of EC, particularly its low surface energy, which imparts hydrophobicity. When hydrophobic EC fibers were placed on water–oil mixtures, they rapidly absorbed motor oil (stained with red dye) within 2 s (Figure 6C, Movie S1). After absorption, the retention stability of the oil in the fibers became a key factor. To test this, the oil-absorbed EC fibers were fully submerged in water using pressure, with buoyancy used to assess the stability of the absorbed oil. The results showed no oil leakage from the EC fibers over an extended period, indicating highly stable oil retention in the porous fibers (Figure 6D, Movie S2).

Recycling ability and cost-effectiveness are critical factors in determining the feasibility and practicality of absorbent materials. EC fibers offer excellent recyclability through simple suction filtration (Figure 6E), making them highly suitable for large-scale waste oil separation applications. The study first evaluated the absorption capacity of EC fibers for various oils, quantified by the weight gain ratio (Q_e_)—the ratio of the absorbed oil mass to the original dry mass of the fibers. The results show that EC fibers demonstrate exceptional oil absorption capacities, absorbing between 56.6 and 110.7 times their own weight, surpassing many previously reported oil-absorbing materials.^51−53^ Notably, fibers with a porous structure, produced at higher flow rates, exhibited a higher oil absorption capacity compared with those produced at lower flow rates (Figure 6F). This increase is attributed to the porous structure, which enhances the surface area and empty space of the fibers, providing more sites for absorption. The pores also facilitate oil penetration into the inner surfaces of the fibers, allowing both the outer and inner surfaces to contribute to the overall absorption, thus increasing efficiency. The absorption capacity of EC fibers varies depending on the type of oil, in the order of motor oil > silicone oil > paraffin oil > cooking oil. This variation can be explained by differences in oil viscosity, where higher-viscosity oils, such as motor oil, are absorbed more effectively than less viscous oils. High-viscosity liquids adhere more strongly to the outer surface of the fibers due to greater cohesion and adhesion forces.^54^

The regeneration of EC fibers through filtration was tested over 10 absorption cycles, and the fibers retained 56% of their saturated absorption capacity after the 10th cycle (Figure 6G). After the first two cycles, the absorption capacity decreased by 44%, primarily due to mechanical compression during filtration, which likely caused the collapse of the porous structure. This structural collapse, driven by intermolecular hydrophobic forces, permanently reduces pore size and volume, limiting the fibers’ ability to absorb oil in subsequent cycles. This behavior contrasts with the more stable absorption capacity of solid-structured EC fibers, which maintain a consistent performance over multiple cycles. Another contributing factor to the reduced absorption in porous EC fibers is the potential clogging of pores with residual oil after initial absorption. Even after filtration, some oil may remain trapped, further reducing the available surface area and pore volume for future absorption cycles. Structural reinforcement or surface modification can be considered in the future to strengthen fiber integrity and protect porosity through multiple cycles, helping to preserve the fibers’ absorptive properties with repeated use. Despite these limitations, the porous EC fibers still exhibited a competitive absorption capacity of 61.8 wt/wt after 10 cycles.

## Conclusions

This study demonstrates significant advancements in the ultrahigh-throughput fabrication of EC fibers with tunable porosity using GUHS-ES. Through the reshaping of the Taylor cone via gravitational forces, this optimized uniaxial electrospinning technique has achieved production rates and structural control that cannot be attained through conventional electrospinning methods. The stabilization and modification of the Taylor cone are essential for this technology’s success. Unlike traditional setups, where the Taylor cone remains hemispherical with a small tip and a high risk of clogging, GUHS-ES shows that as the flow rate increases from 1 to 150 mL/h, the Taylor cone retracts into the needle, and the tip expands outward. This reshaped Taylor cone supports high flow rates, dramatically increasing EC fiber production to 24.5 g/h—hundreds of times higher than conventional electrospinning yields. GUHS-ES offers a dual benefit: higher throughput and improved fiber porosity for specific applications (e.g., oil absorption). By adjustment of the flow rate, the diameter uniformity and porosity of the fibers can be finely controlled. At lower flow rates (1–10 mL/h), the EC fibers exhibit solid structures with beaded morphology and uneven diameters. At increased flow rates (20–60 mL/h), the stretching force removes most beads, slightly thickens the fibers, and promotes uniformity. At ultrahigh flow rates (100–150 mL/h), fibers are homogeneous with stable diameters, and the average pore size reaches a maximum of 321 nm at 150 mL/h. BET analysis shows a significant increase in the surface area as flow rates rise, from 5.91 m^2^/g at 1 mL/h to 16.89 m^2^/g at 150 mL/h. These porous EC fibers demonstrate superior oil absorption capacity, absorbing up to 120 times their weight in various oils and significantly outperforming most existing materials. Additionally, the compressibility of the EC fibers allows for efficient oil recovery through filtration, greatly reducing the material’s operational costs. Our findings challenge the conventional limits of electrospinning and open new avenues for the efficient, scalable production of fibers with tailored properties. In summary, the GUHS-ES technology offers a green, efficient method for producing EC fibers with tunable porosity and excellent oil absorption capacity. This technology has strong potential to advance EC fiber production and support widespread applications in diverse fields.

## Supporting Information Available

SEM images illustrating the morphology, surface structure, and internal structure of EC fibers produced in a vertical setup at various flow rates; additionally, SEM images display the morphology and surface structure of EC fibers fabricated in a horizontal setup at different flow rates; further details include BJH desorption pore size distribution curves and photographs showing water and oil contact angles for solid-structured EC fibers (PDF)
Demonstration of porous EC nanofibers quickly and effectively absorbing oil (MP4)
Demonstration of the stability of oil-absorbed EC nanofibers, showing no oil leakage when fully submerged in water (MP4)
