# Supplementary material for: Gravity-Driven Ultrahigh-Speed Electrospinning for the Production of Ethyl Cellulose Fibers with Tunable Porosity for Oil Absorption
Source: ACS Sustain Chem Eng. 2024 Dec 19;13(1):507–17. doi: 10.1021/acssuschemeng.4c08259 (PMC11734104; doi:10.1021/acssuschemeng.4c08259)
Supplement: Supplementary file 1 — sc4c08259_si_001.pdf [file sc4c08259_si_001.pdf]

Supporting Information

For

Gravity-Driven Ultrahigh-Speed Electrospinning for Production of Ethyl Cellulose Fibers with Tunable Porosity for Oil Absorption

Qiangjun Hao,[1] John Schossig,[1] Tyler Davide,[2] Adedayo Towolawi,[1] Cheng Zhang,[2] and Ping Lu[1,*]

[1] Department of Chemistry and Biochemistry, Rowan University, Glassboro, New Jersey 08028, United States

[2] Chemistry Department, Long Island University (Post), Brookville, NY 11548, United States

*Address correspondence to lup@rowan.edu (P. Lu).

Number of pages: 10

Number of figures: 7

Number of movies: 2

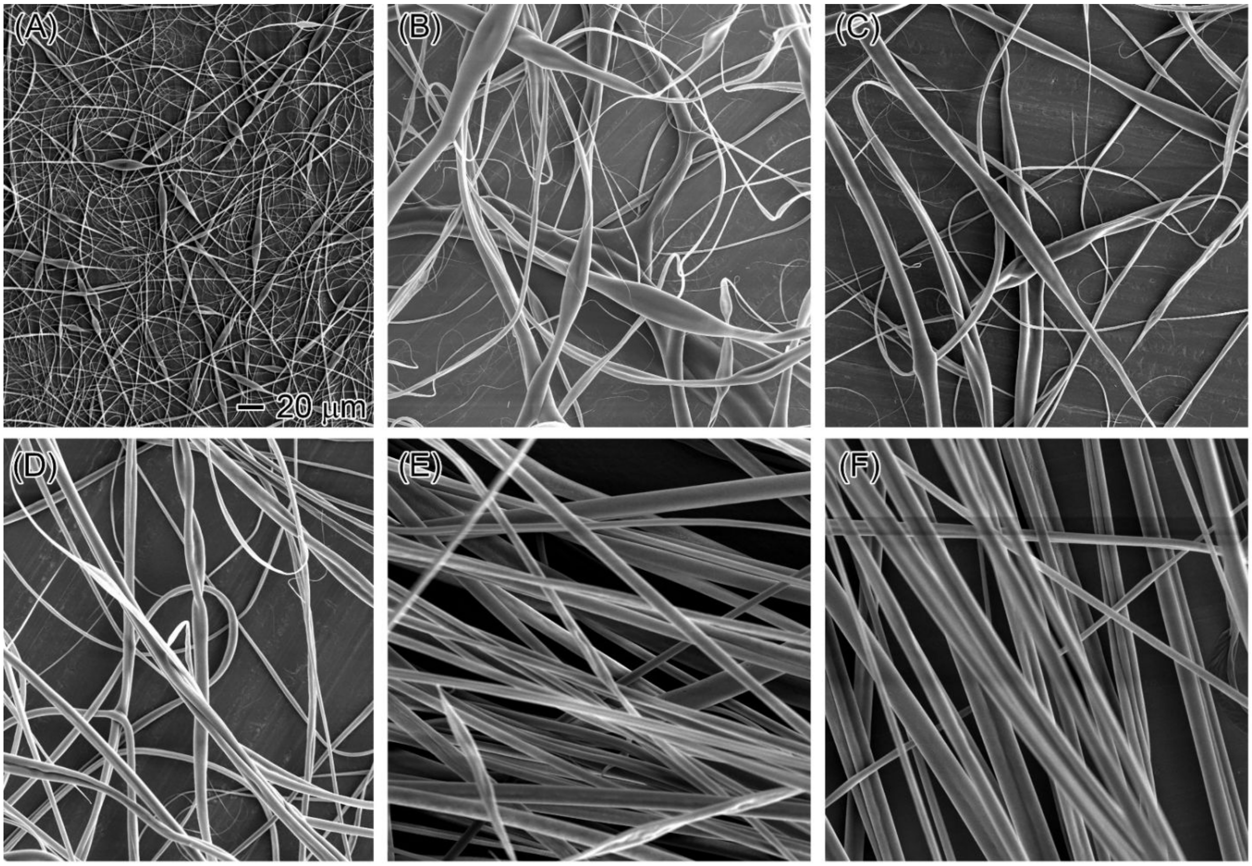

Figure S1. SEM images showing the morphology of EC fibers at various flow rates: (A) 10 mL/h, (B) 20 mL/h, (C) 40 mL/h, (D) 50 mL/h, (E) 60 mL/h, and (F) 100 mL/h. The scale bar in (A) applies to all images.

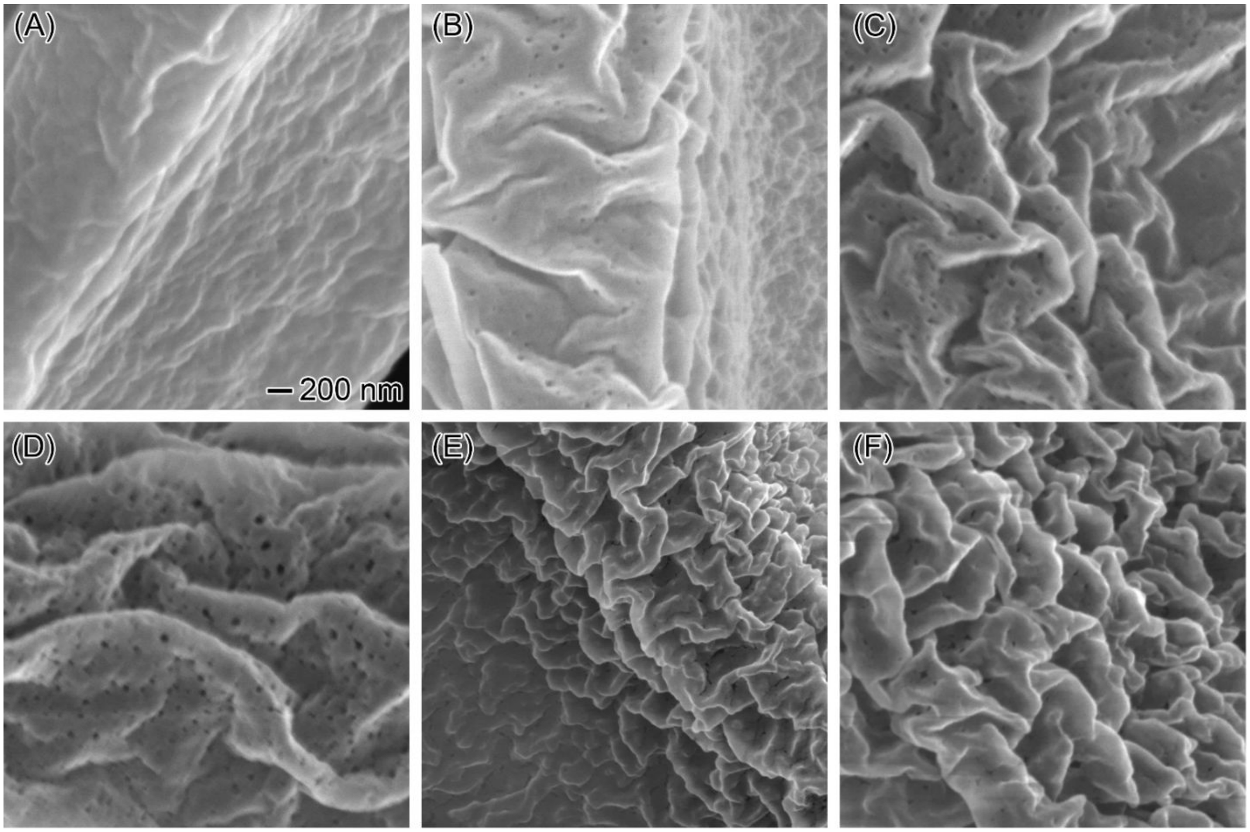

Figure S2. SEM images showing the surface morphology of EC fibers at various flow rates: (A) 10 mL/h, (B) 20 mL/h, (C) 40 mL/h, (D) 50 mL/h, (E) 60 mL/h, and (F) 100 mL/h. The scale bar in (A) applies to all images.

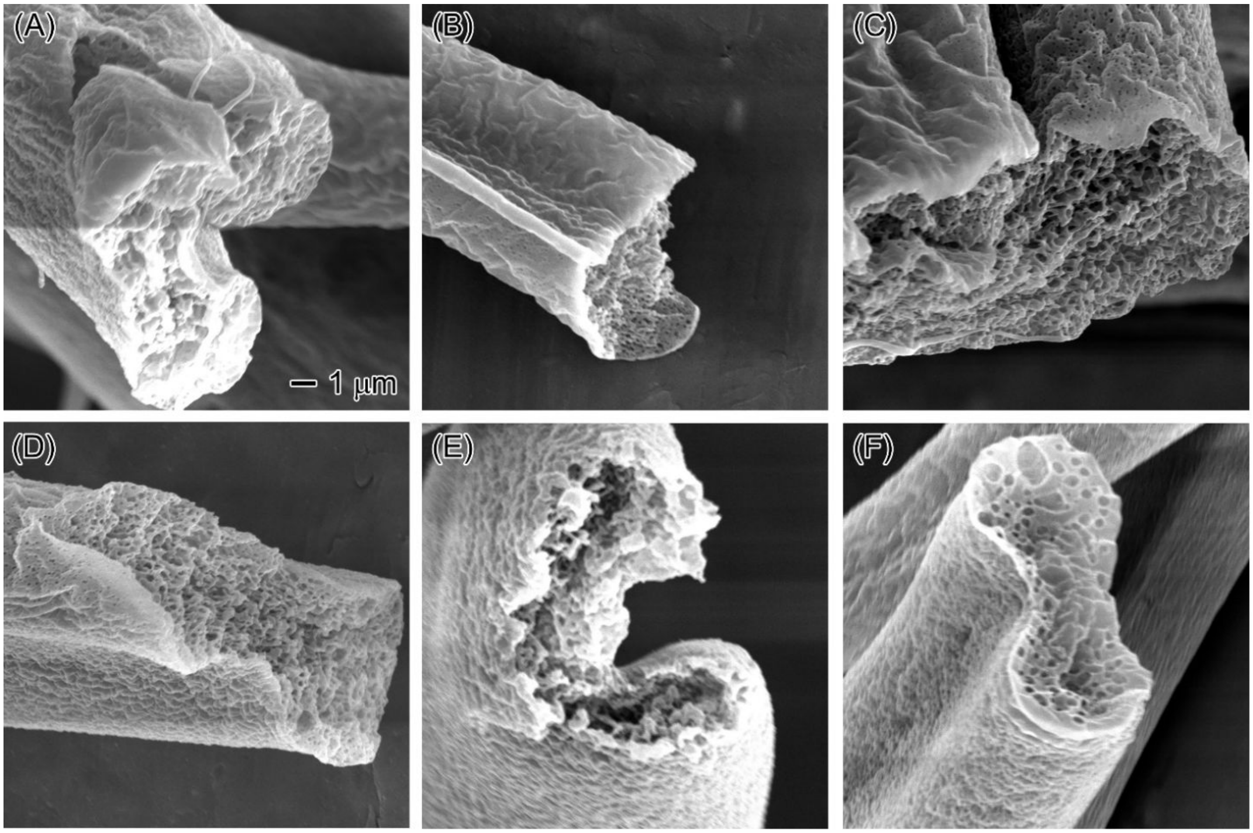

Figure S3. SEM images showing the porosity of EC fibers at various flow rates: (A) 10 mL/h, (B) 20 mL/h, (C) 40 mL/h, (D) 50 mL/h, (E) 60 mL/h, and (F) 100 mL/h. The scale bar in (A) applies to all images.

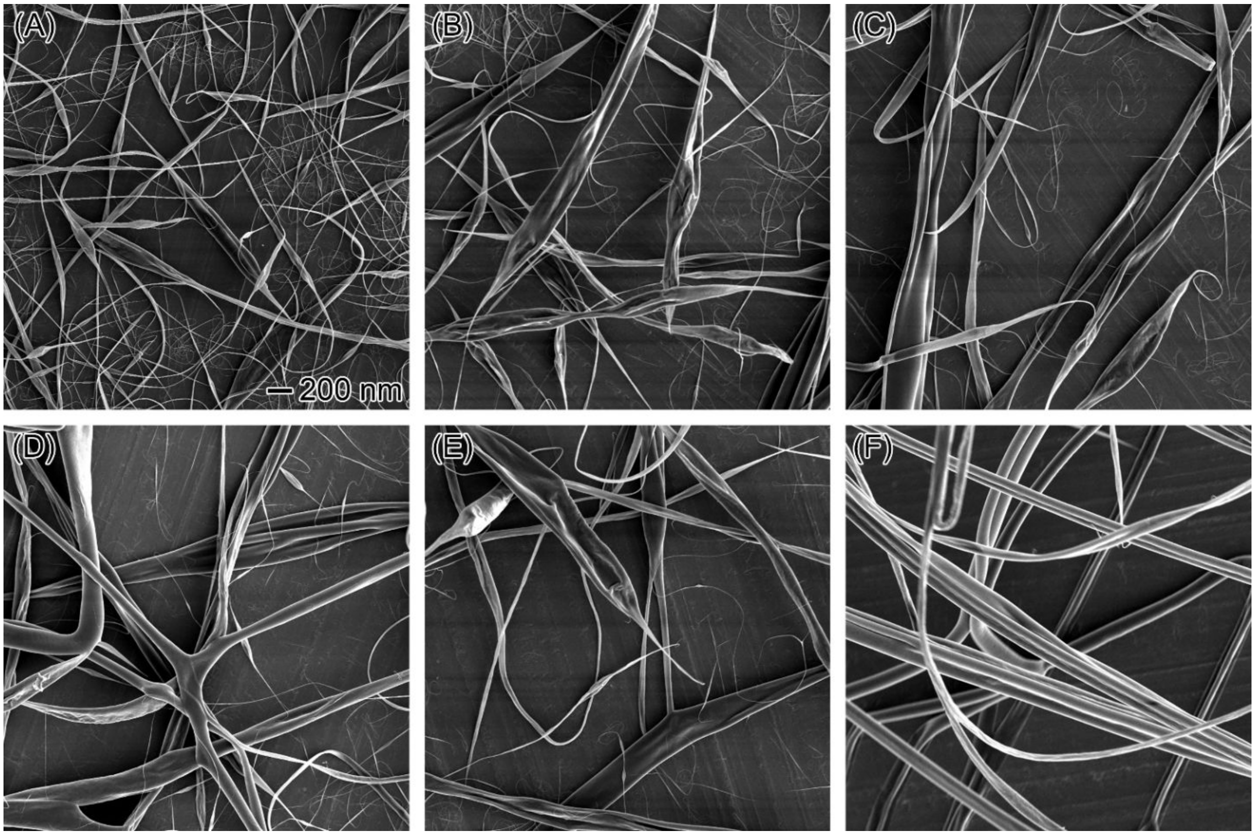

Figure S4. SEM images showing the morphology of EC fibers in a horizontal setup at various fl ow rates: (A) 10 mL/h, (B) 20 mL/h, (C) 40 mL/h, (D) 50 mL/h, (E) 60 mL/h, and (F) 100 mL/h. The scale bar in (A) applies to all images.

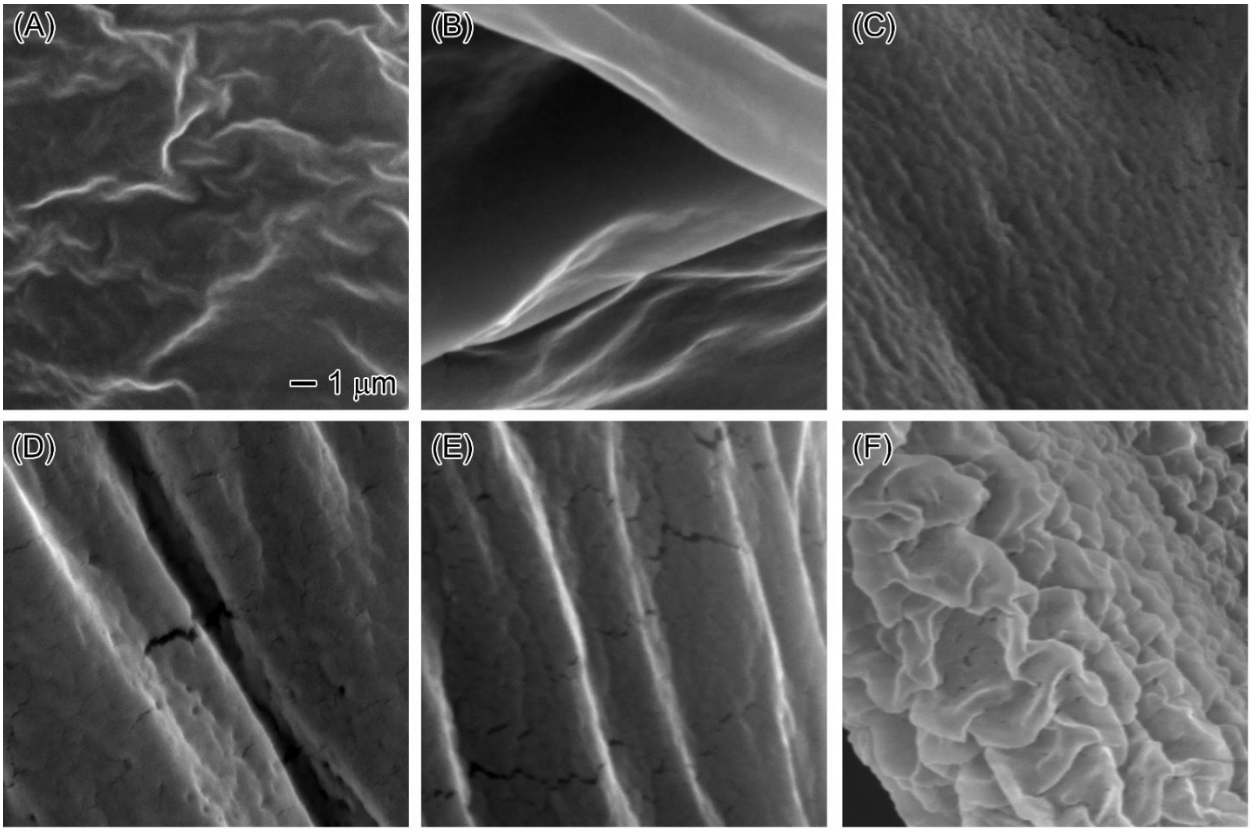

Figure S5. SEM images showing the surface roughness of EC fibers in a horizontal setup at diffe rent flow rates: (A) 10 mL/h, (B) 20 mL/h, (C) 40 mL/h, (D) 50 mL/h, (E) 60 mL/h, and (F) 100 mL/h. The scale bar in (A) applies to all images.

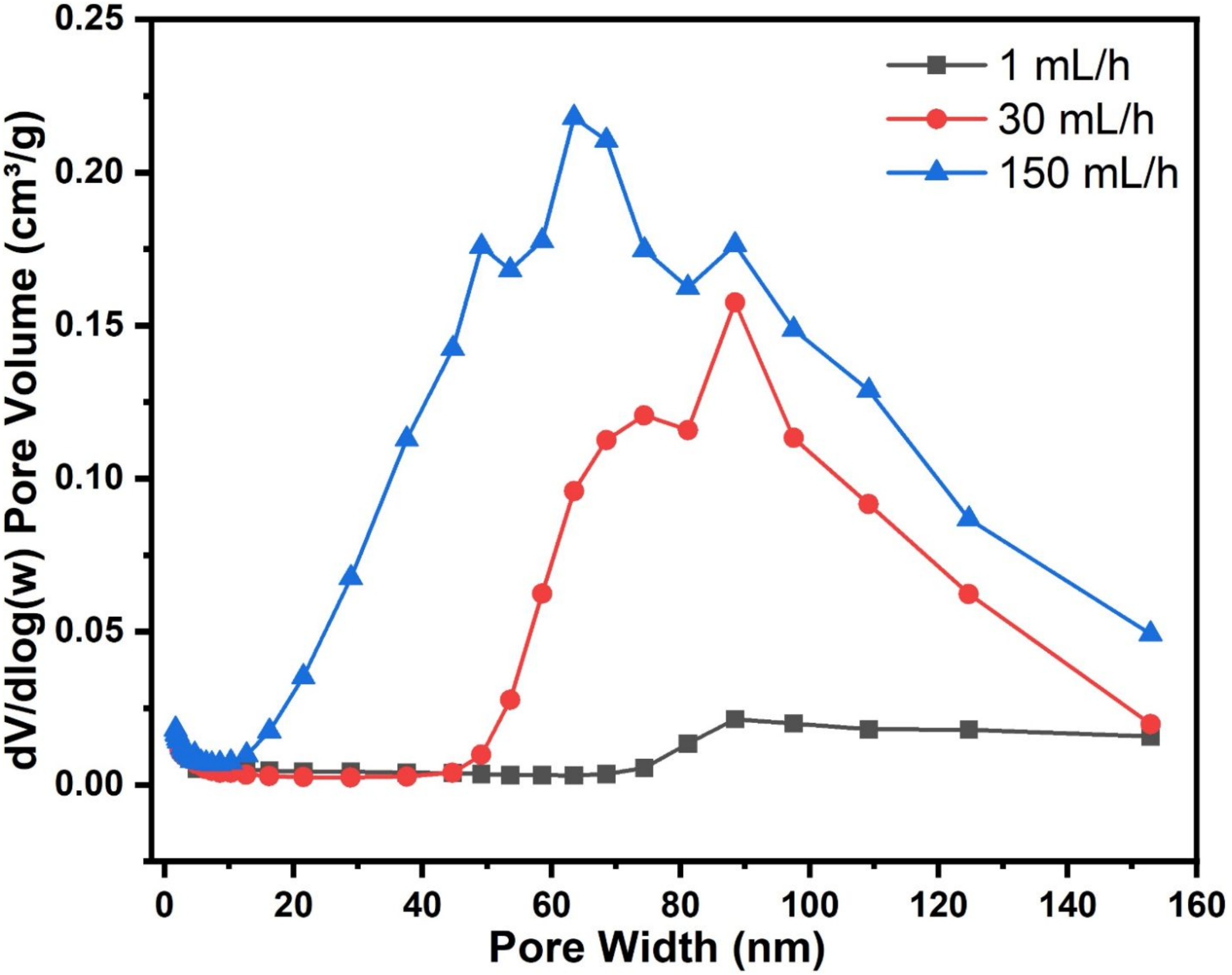

Figure S6. BJH desorption pore size distribution curves for electrospun EC nanofibers at different flow rates (1 mL/h, 30 mL/h, and 150 mL/h). The x-axis represents pore width (nm), while the y-axis represents pore volume (cm^3/g). These curves illustrate how flow rate influences the pore size distribution and pore volume of the nanofibers.

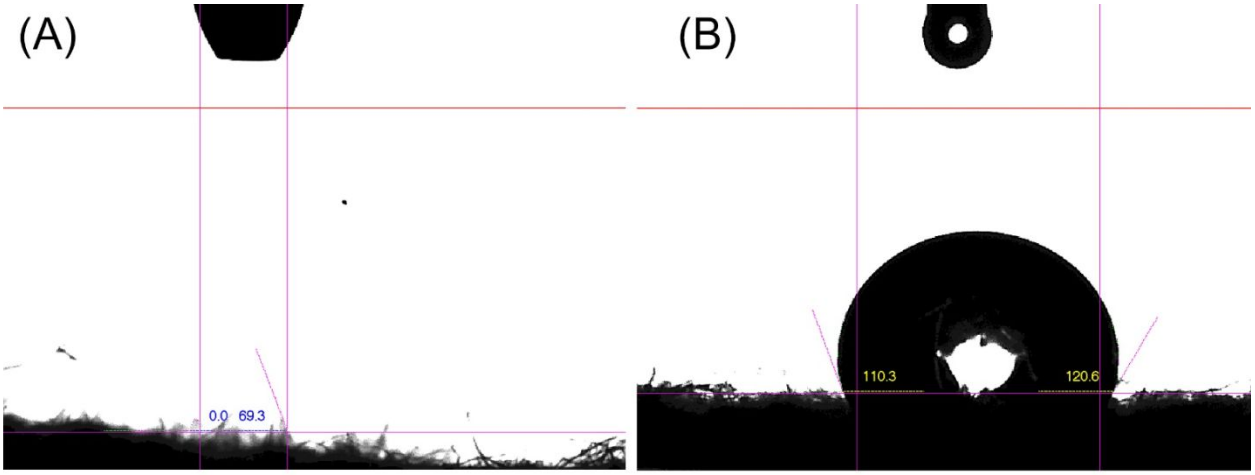

Figure S7. Oil (A) and water (B) contact angles of solid-structured EC nanofibers produced at a flow rate of 1 mL/h, showing the hydrophobic and oleophilic properties of the fibers.

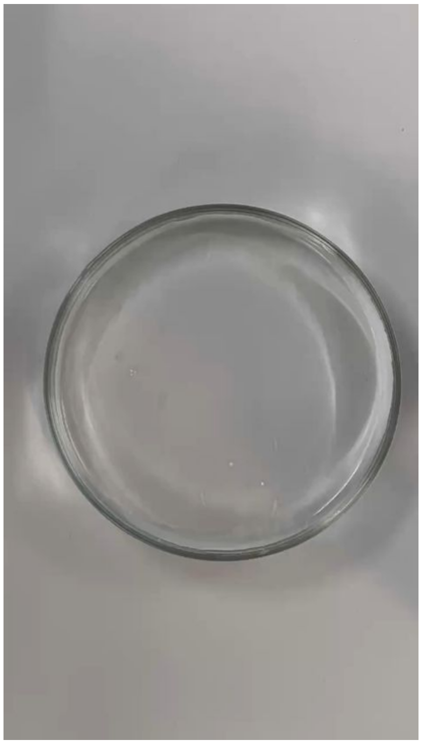

Movie S1. Demonstration of porous EC nanofibers quickly and effectively absorbing oil.

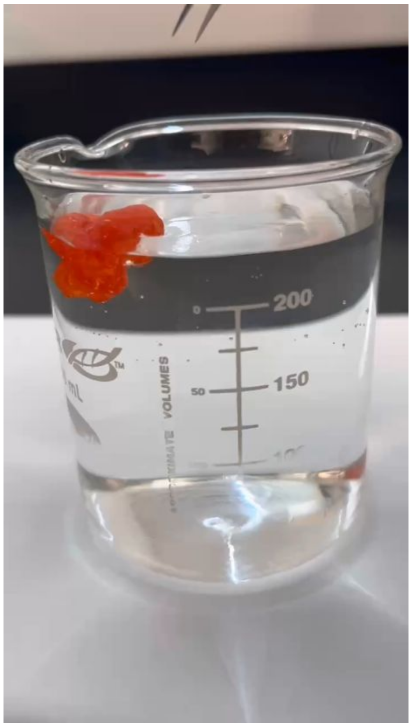

Movie S2. Demonstration of the stability of oil-absorbed EC nanofibers, showing no oil leakage when fully submerged in water.
